# Congenital Absence of the Internal Carotid Artery Associated With Contralateral Middle Cerebral Artery Occlusion in a Young Female Patient: Embryology, Multimodal Imaging Findings, and Clinical Implications

**DOI:** 10.7759/cureus.91067

**Published:** 2025-08-26

**Authors:** Juan Martin Leguízamo-Isaza, Laura Olarte Bermúdez, Sofía Catalina Velasco-Sandoval, Juan Andrés Mejía-Nuñez

**Affiliations:** 1 Radiology, Fundación Santa Fe de Bogotá, Bogotá, COL

**Keywords:** circle of willis, collateral circulation, congenital disease, internal carotid artery, stroke

## Abstract

Congenital absence of the left internal carotid artery (ICA) is a rare condition associated with contralateral occlusion of the right middle cerebral artery (MCA) in a young female patient, highlighting the imaging findings and clinical characteristics of this anatomical variation. We describe the embryological basis, clinical presentation, multimodal imaging findings, and the relevance of collateral circulation development in a patient with a left congenital absence of the ICA associated with contralateral MCA occlusion. In this case, we present a 32-year-old woman with signs and symptoms compatible with a right MCA stroke. The initial multimodal imaging workup revealed an absent left ICA and a left common carotid artery originating from the arterial brachiocephalic trunk. This case highlights a thorough and updated perspective of developmental ICA congenital absence associated with contralateral MCA occlusion, emphasizing key differentiators from other acute cerebrovascular conditions and the potential clinical implications of this anatomical variant.

## Introduction

Congenital absence of the internal carotid artery (ICA) is a rare vascular anatomical variant characterized by either complete absence or underdevelopment of the ICA. It results from anomalies in the third aortic arch or the dorsal aorta during early embryogenesis, with a reported prevalence of fewer than 10 cases [1,2]. ICA congenital absence can be unilateral or bilateral [3]. It is usually asymptomatic and incidentally detected due to sufficient collateral circulation via the circle of Willis.

Under normal conditions, the ICA originates from the common carotid artery (CCA), traverses the skull base through the carotid canal, and supplies the anterior cerebral circulation, including the middle cerebral artery (MCA) and anterior cerebral artery (ACA). Awareness of this anatomy is fundamental to understanding the clinical implications of its congenital absence. Although congenital absence of the ICA is exceedingly rare, when this anomaly coexists with contralateral MCA occlusion, this combination imposes a unique hemodynamic burden.

Diagnosis of this anomaly is pivotal, as it is associated with an increased risk of cerebral aneurysms and intercavernous anastomoses, requiring additional planning before procedures such as carotid or transsphenoidal surgery in the context of thromboembolic disease and its surveillance [1,3]. In our literature review, we found fewer than 10 case reports of ICA absence associated with contralateral MCA occlusion. This combination is exceptionally rare and poses a unique hemodynamic burden. We describe a case with a highly atypical clinical presentation in which various other differential diagnoses were initially considered. This report emphasizes the relevance of a multimodal diagnostic imaging approach, the key imaging findings in this entity, and the differential diagnoses to consider. 

## Case presentation

A 32-year-old woman with a history of ischemic stroke one year prior in the left MCA territory. At that time, vascular imaging had demonstrated a moderate-to-severe occlusion (approximately 50%) of the right MCA, which resulted in residual right hemiparesis and speech disturbances. She presented to the emergency department two hours after experiencing altered consciousness and bilateral palpebral ptosis. At admission, her National Institutes of Health Stroke Scale (NIHSS) score was 10, calculated for the current presentation and excluding residual deficits from the prior stroke without clear focalization. Prodromal symptoms included headache, nausea, and vomiting.

**Figure 1:**
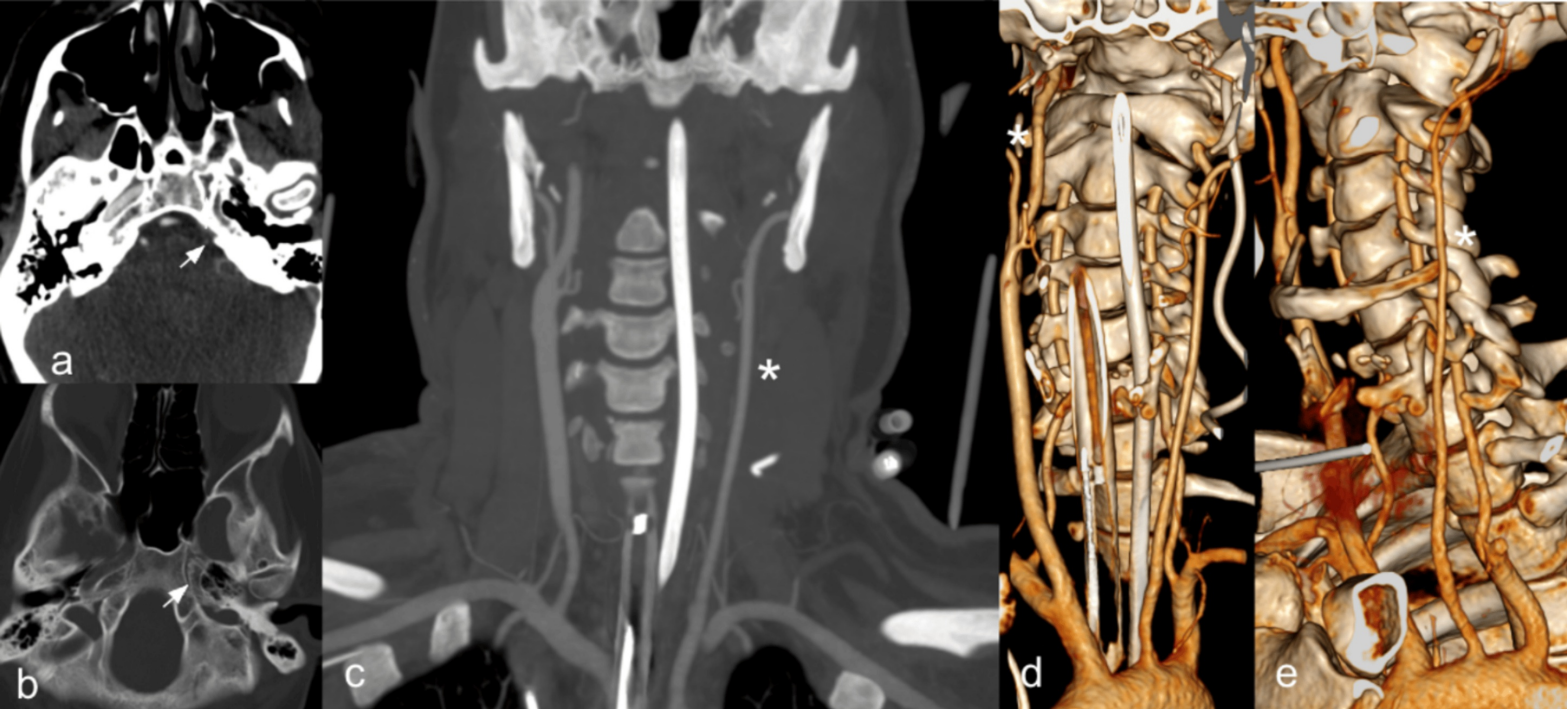
CTA of the head and neck Images in the axial (a, b) and coronal (c) plane, as well as coronal 3D reconstructions (d, e) demonstrating congenital absence of the left ICA and left carotid canal (white arrow). The left CCA can be seen originating from the aortic arch and giving rise to the left external carotid artery (*). ICA: internal carotid artery; CCA: common carotid artery

Physical examination revealed altered consciousness without tonic-clonic movements or sphincter relaxation. Given these clinical findings, code stroke was activated, and the patient was ordered a computed tomography angiography (CTA) given her history of a prior stroke. The images demonstrated occlusion of the distal portion of the M1 segment of the right MCA and showed that the left common carotid artery (CCA) had a conventional origin from the aortic arch, but lacked bifurcation, continuing as the left external carotid artery (ECA) and its branches, which were patent. In addition to the absence of the ICA, the ipsilateral carotid canal at the skull base was also absent, findings compatible with developmental congenital absence (Figure 1). 

**Figure 2:**
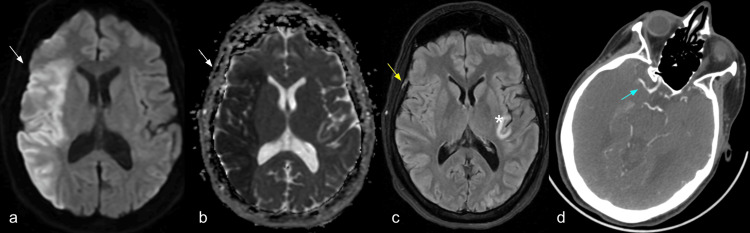
Brain magnetic resonance imaging study Images showing changes that indicate restricted diffusion in the vascular territory of the right middle cerebral artery (white arrows), without any representation on the T2/ FLAIR images (yellow arrow), findings in keeping with acute infarction. Additionally, the left insular region showed changes compatible with subacute infarction (asterisk) and occlusion of the distal portion of the M1 segment of the right middle cerebral artery (blue arrow).

**Figure 3:**
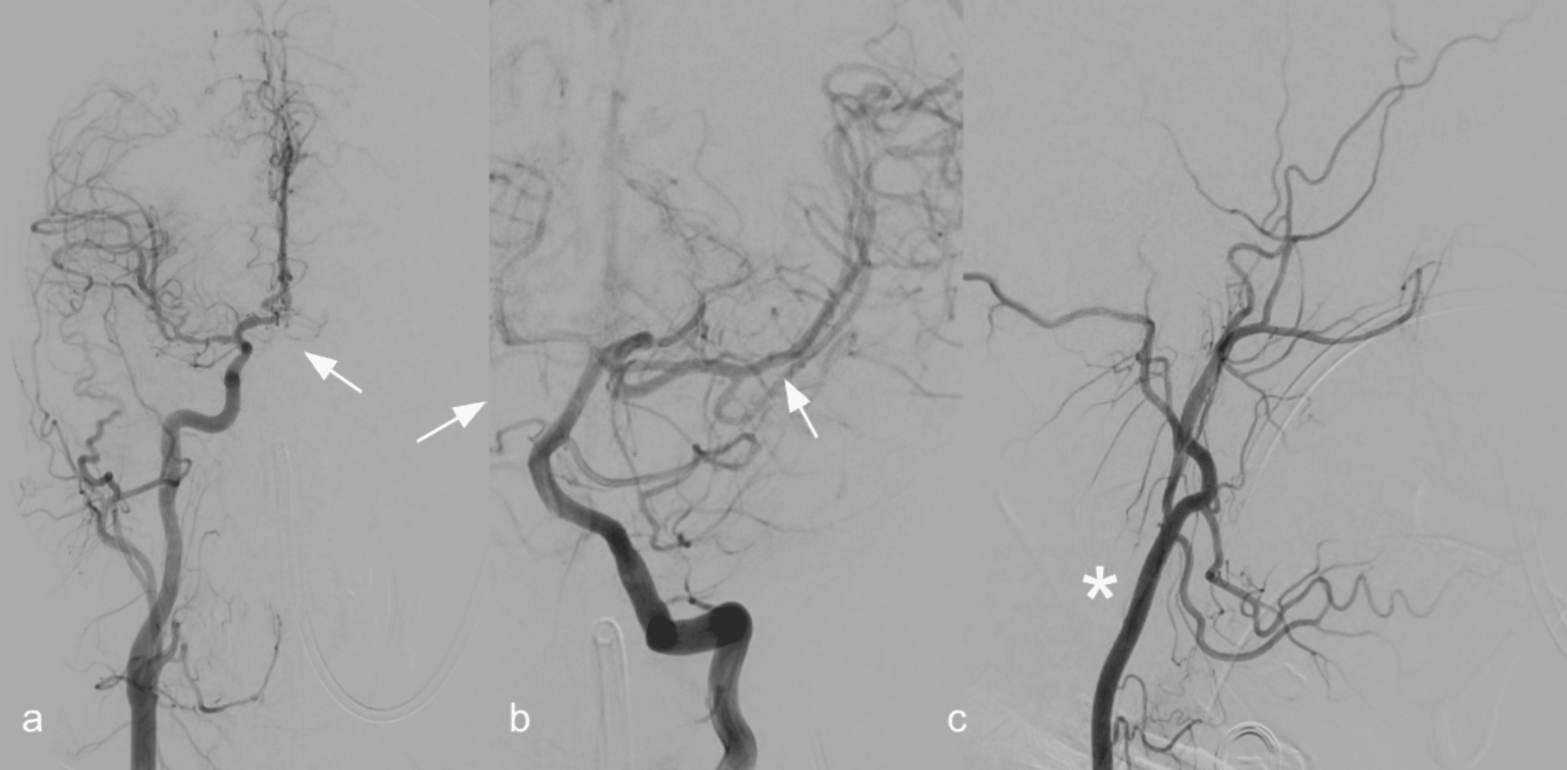
Cerebral digital subtraction angiography (DSA) (a-c) (a) Frontal view of a right-sided CCA injection demonstrating opacification of segment A2 of the anterior cerebral artery (ACA) (white arrow) through the anterior communicating artery (Acom). (b) Oblique view of a left vertebral artery (VA) injection showing opacification of the ipsilateral MCA (white arrow) via the posterior communicating artery (PComA). (c) Frontal view of a left ECA injection (*) depicting normal opacification of its branches. MCA: middle cerebral artery; ECA: external carotid artery; CCA: common carotid artery

Subsequently, and nearly one hour after the brain CTA was performed, the patient underwent a code stroke abbreviated magnetic resonance imaging (MRI) study, which included axial fluid-attenuated inversion recovery (FLAIR) and diffusion-weighted imaging (DWI) sequences (Figure 2). The main findings were extensive diffusion restriction of about two-thirds of the right MCA territory, without any FLAIR abnormalities, which is in keeping with diffusion/FLAIR mismatch and suggested acute infarction. For these reasons, both vascular neurology and interventional neuroradiology agreed to perform panangiography (Figure 3). The patient was managed with an endovascular mechanical thrombectomy, which served both diagnostic and therapeutic purposes and successfully resulted in the extraction of a floating thrombus at the level of the communicating segment of the right ICA. A coagulopathy, most likely thrombophilia, was concluded to be the underlying etiology, and so the patient was initiated on warfarin therapy.

## Discussion

Congenital absence of the ICA is a rare condition characterized by either complete absence or underdevelopment of the ICA. The definition of "absence" includes agenesis, aplasia, and hypoplasia of the ICA, conditions that may often be asymptomatic due to extensive collateral circulation via the circle of Willis [1]. During embryogenesis, aortic development begins with ventral and dorsal segments and six paired primitive aortic arches in which the primitive third aortic arch gives origin to the CCA and the cervical segment of the ICA, while the circle of Willis is formed between 7 and 24 mm embryonic stages. The mechanism of congenital absence of the ICA is due to stress (amniotic bands) or excessive bending of the cephalic segment of the embryo during development [4,5]. 

Collateral circulation, mainly via the circle of Willis, through the anterior communicating artery and posterior communicating artery, is essential for maintaining adequate cerebral perfusion in these cases and plays a determinant role in the patient’s prognosis. The primary compensatory pathways include the anterior and posterior communicating arteries, as well as the ophthalmic arteries. The effectiveness of these collateral routes varies among patients and influences the risk of ischemic events taking place. In this case, collateral flow via anterior cerebral circulation was insufficient to prevent ischemia, especially in the presence of significant contralateral right MCA occlusion. The compensatory demand on the remaining vasculature may contribute to turbulent flow and increased shear stress, predisposing to aneurysm formation and progressive occlusion disease [6]. 

Given the rarity of this presentation, patients with ICA congenital absence face an elevated risk of cerebrovascular disease, particularly when contralateral occlusion is present. Hemodynamic imbalance in conjunction with other factors reduces the blood supply and can lead to tissue damage, chronic hypoperfusion, and increased susceptibility to thromboembolic events. For that reason, in the context of thromboembolic disease, it is important to recognize this anomaly because emboli found in one cerebral hemisphere may be explained by atherosclerotic disease in the contralateral right CCA or the vertebrobasilar system [1,7].

Moreover, radiologists must evaluate other intracranial vascular anomalies typically associated with ICA agenesis. Between 25%-34% of these patients have been found to have associated cerebral aneurysms [6]. In contrast to typical cases of ICA congenital absence, contralateral occlusion of the MCA has not been extensively studied in this setting.

Multimodal imaging plays a fundamental role in the diagnosis. Most cases are initially identified via ultrasonography, MRI, or computed tomography (CT), depending on the clinical presentation. One critical aspect to consider is the presence of the ICA or its precursor for the development of the carotid canal. CT is the primary imaging modality for differentiating ICA aplasia from congenital absence by assessing the presence or absence of the carotid canal. This allows assessment of the bony pathway of the ICA through the skull base and cavernous sinus [8]. Cerebral digital subtraction angiography is then necessary to confirm the diagnosis, demonstrating an anomalous origin of the ophthalmic artery, which may arise from the ipsilateral middle meningeal artery or the proximal segment of the MCA [9]. MRI provides further characterization of vascular structures and collateral circulation patterns.

In management, the congenital absence of ICA has implications for planning procedures such as carotid endarterectomy, transsphenoidal pituitary surgery and endovascular interventions.

The congenital absence of the ICA has a significant impact on surgical and endovascular planning. In a possible future case where the lesion is ipsilateral to the congenital absence, the procedure could require access via the contralateral ICA through the anterior communicating artery, making pre-procedural imaging essential for route planning and patient safety. Therefore, procedures such as carotid endarterectomy and transsphenoidal pituitary surgery are contraindicated on the affected side because of the absent collateral flow, increasing the risk of ischemia in case of complications. Additionally, the loss of balance between intra- and extracranial circulation disrupts cerebral autoregulation, heightening stroke risk, especially when contralateral MCA occlusion is present.

In this patient, the degree of right MCA occlusion (approximately 50%) represents a major risk factor for recurrent ischemic events and must be carefully monitored to avoid progression with devastating consequences. Management of intracranial atherosclerotic disease typically includes antiplatelet therapy; however, in this case, the concomitant indication for anticoagulation due to suspected thrombophilia posed a therapeutic challenge.

Overall, the prognosis is highly variable depending on whether the finding was incidental or secondary to a more specific set of signs and symptoms. Currently, there is no exclusive therapy for congenital absence of the ICA. However, this case underscores the need for symptomatic management and reconstruction of contralateral circulation pathways or when it is associated with cerebral aneurysms. The patient underwent successful mechanical thrombectomy and required acute-phase rehabilitation, including physical therapy and speech therapy. She was initiated on anticoagulant and statin therapy and showed favorable clinical recovery, with significant improvement observed at the two-month follow-up.

## Conclusions

In conclusion, congenital absence of the ICA associated with contralateral occlusion is a rare entity that presents unique diagnostic challenges. Although often undetected for long periods of time due to extensive collateral circulation, symptomatic cases associated with ischemic stroke require timely recognition and urgent management. Awareness of anatomical variants and their clinical associations is paramount for establishing an accurate diagnosis and making appropriate clinical decisions. Clinicians should maintain a high index of suspicion and consider vascular imaging in atypical stroke presentations, particularly in younger patients without traditional risk factors. Imaging surveillance should be considered in patients with cerebrovascular symptoms or other vascular anomalies to identify similar congenital conditions. Nonetheless, these conclusions are based on a single patient and thus have limited generalizability, serving primarily as hypothesis-generating observations.
